# Intracellular Flux Prediction of Recombinant *Escherichia coli* Producing Gamma-Aminobutyric Acid

**DOI:** 10.4014/jmb.2312.12022

**Published:** 2024-01-30

**Authors:** Sung Han Bae, Myung Sub Sim, Ki Jun Jeong, Dan He, Inchan Kwon, Tae Wan Kim, Hyun Uk Kim, Jong-il Choi

**Affiliations:** 1 Department of Chemical and Biomolecular Engineering, Korea Advanced Institute of Science and Technology (KAIST), Daejeon 34141, Republic of Korea; 2 Department of Biotechnology and Bioengineering, Chonnam National University, Gwangju, 61186, Republic of Korea; 3 College of Life Science and Agriculture Forestry, Qiqihar University, Qiqihar, 161006, Heilongjiang, China; 4 School of Materials Science and Engineering, Gwangju Institute of Science and Technology, Gwangju 61005, Republic of Korea

**Keywords:** Genome-scale metabolic model, gamma-aminobutyric acid, fermentation, *Escherichia coli*

## Abstract

Genome-scale metabolic model (GEM) can be used to simulate cellular metabolic phenotypes under various environmental or genetic conditions. This study utilized the GEM to observe the internal metabolic fluxes of recombinant *Escherichia coli* producing gamma-aminobutyric acid (GABA). Recombinant *E. coli* was cultivated in a fermenter under three conditions: pH 7, pH 5, and additional succinic acids. External fluxes were calculated from cultivation results, and internal fluxes were calculated through flux optimization. Based on the internal flux analysis, glycolysis and pentose phosphate pathways were repressed under cultivation at pH 5, even though glutamate dehydrogenase increased GABA production. Notably, this repression was halted by adding succinic acid. Furthermore, proper *sucA* repression is a promising target for developing strains more capable of producing GABA.

## Introduction

Gamma-aminobutyric acid (GABA) is a non-proteinogenic amino acid with extensive commercial applications. It is often used as a food supplement or drug due to its physiological and pharmacological functions, such as lowering blood pressure, preventing diabetes, relieving mental stress or anxiety, and treating epilepsy [1-4]. GABA is also a 2-pyrrolidone precursor, a monomer of nylon 4, which can be used for engineering plastic with high heat resistance and biodegradability [5]. Such versatility has led to many studies aiming to improve GABA production through chemical or biological processes. However, due to the environmental problems such as global warming and fossil oil depletion by chemical synthesis process, most attempts focus on improving sustainability and efficiency using microorganisms [4, 6, 7]. Representative microbial hosts for GABA production include *Saccharomyces cerevisiae* [8], *Bacillus megaterium* [7], *Monascus* species [9], and *Escherichia coli* [6, 10-17]. Thus far, *E. coli* has been the most widely used microbial host for GABA production because of its high growth rate and well-developed genetic tools. There are two primary strategies for producing GABA using *E. coli* as a host: (1) the decarboxylation of monosodium glutamate (MSG) or L-glutamate [6, 11-14, 18] in the GABA shunt pathway or (2) fermentative GABA production from glucose or other carbon sources [15-17]. For glutamate decarboxylation, GABA production has been improved by overexpressing the native glutamate decarboxylase (GAD), introducing heterologous GAD into *E. coli*, or engineering the coenzyme factor pyridoxal 5-phosphate (PLP). However, a fermentation process for glutamate production is required for glutamate to be converted into GABA through enzyme or whole-cell reactions. Therefore, a one-step fermentation method using carbon sources was investigated for simple and economical GABA production. Several studies have attempted to engineer a central carbon metabolism associated with GABA production in *E. coli*. For example, co-localization of glutamate synthase, the GAD and GABA transporter [17], and synthetic scaffolds that connect isocitrate dehydrogenase, glutamate synthase, and GAD [15] have been implemented to improve GABA production from glucose. More recently, DR1558, a response regulator from *Deinococcus radiodurans*, was successfully introduced into *E. coli*, improving cellular tolerance to external stresses, including low pH. This reaction is because GAD’s optimal pH is around 4.5, while *E. coli* fermentation is conducted near 7.0 [19]. In particular, DR1558 expression in recombinant *E. coli* was interesting because of the potentially high GABA productivity during cell growth.

**Fig. 1:**
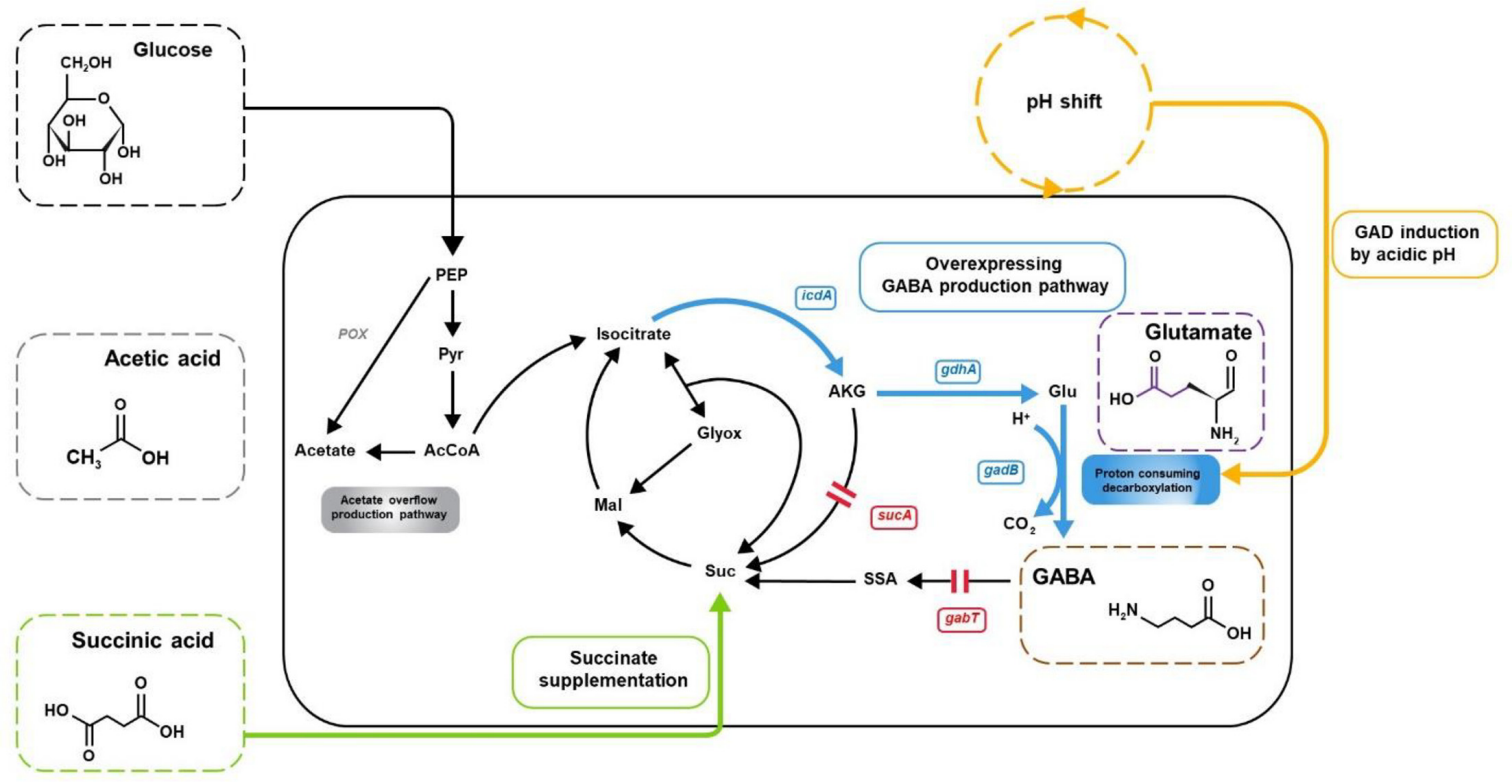
Construction of the GABA production pathway to directly produce GABA from glucose. The blue terms represent gene overexpression. The red terms represent the knockout of genes.

This study investigated GABA production using *E. coli* strains harboring DR1558, and metabolic flux distributions under various culture conditions were calculated with a genome-scale metabolic model (GEM). In particular, we examined the effects of the pH drop and adding succinic acid in the metabolic phenotypes of *E. coli* strains (Fig. 1). GEM is a computational model containing information on all metabolic genes in a cell and can be simulated for the cellular metabolic phenotypes under various environmental or genetic conditions [20, 21]. We obtained further insight into metabolic perturbation in the *E. coli* host induced by GABA-producing conditions and predicted the next engineering targets for future work by identifying bottleneck reactions.

## Materials and Methods

### Bacterial Strains and Plasmids

In this study, *E. coli* DGB303 was used as a GABA-producing strain constructed by transforming pH3BN and pGB103 into *E. coli* GB300 (BL21(DE3) ***D**gabT**D**sucA*) [19]. pH3BN expressing *Neurospora crassa* *gadB* and *E. coli* *gadC* converted glutamate into GABA. pGB103 was constructed by inserting *D. radiodurans* *dr1558*, *E. coli* *gdhA*, and *E. coli* *icdA* into the pACYCDuet-1 (Novagen, USA) plasmid [19].

### Culture Conditions

*E. coli* DGB303 strains were cultivated in fermenters (MARADO-05S-XS, BIOCNS Co., Republic of Korea). Pre-seed cultures were prepared by inoculating a single colony on a Luria-Bertani (LB) medium (10 g/l tryptone, 5 g/l yeast extract, and 5 g/l NaCl, respectively) agar plate with antibiotics into 15 ml test tubes containing 4 ml LB medium. Then, pre-seed cultures were cultivated in a shaking incubator for 12 h at 30°C and 250 rpm. To prepare seed culture, 1 ml of pre-seed culture was inoculated in a 250 ml flask containing 50 ml MR medium supplied with 20 g/l glucose, 2 g/l succinic acid, 10 g/l ammonium sulfate, and 5 g/l yeast extract.

The MR medium (pH 7.0) composition was as follows: 6.67 g/l KH_2_PO_4_, 4 g/l (NH_4_)_2_HPO_4_, 0.8 g/l MgSO_4_·7H_2_O, 0.8 g/l citric acid, and 5 ml/l of trace metal solution. The trace metal solution composition was as follows: 10 g/l FeSO_4_·7H_2_O, 2 g/l CaCl_2_, 2.2 g/l ZnSO_4_·7H_2_O, 0.5 g/l MnSO_4_·4H_2_O, 1 g/l CuSO_4_·5H_2_O, 0.1 g/l (NH_4_)_6_Mo_7_O_24_·4H_2_O, and 0.02 g/l Na_2_B_4_O_7_·10H_2_O prepared in 0.5 M HCl. MgSO_4_·7H_2_O was sterilized separately. Next, the seed culture was cultivated in a shaking incubator at 30°C and 250 rpm for 12 h. Batch fermentations were performed to produce GABA from glucose at 30°C in a 5 L fermenter (MARADO-05S-XS, BIOCNS Co., Republic of Korea) containing an initial 1.8 L of MR medium supplemented with 20 g/l glucose, 2 g/l or 4 g/l succinic acid, 10 g/l ammonium sulfate, and 5 g/l yeast extract.

Batch culture was initiated by inoculating 200 ml of the seed culture, supplying air at a 2 L/min flow rate, and agitating at 300 rpm. The culture pH was maintained at 6.9 ~ 7.1 by automatically adding 5 M NaOH and 5 M HCl. The dissolved oxygen concentration (DOC) was maintained at 20% of air saturation by automatically adjusting the agitation speed to 1,000 rpm and supplying pure oxygen gas if necessary. When the culture medium’s optical density at 600 nm (OD_600_) was 5.0, isopropyl β-D-1-thiogalactopyranoside (IPTG) was added at a 0.5 mM concentration to induce protein expression. Ampicillin (Ap, 100 μg/ml) and chloramphenicol (Cm, 35 μg/ml), used as antibiotic resistance markers for plasmids, were added to the medium if necessary.

Dual-phase fermentation was also carried out by lowering the pH from 7.0 to 5.0 in the broth during IPTG induction to increase GABA production. This dual-phase cultivation allows the separation of the neutral pH condition (the first phase) for cell growth from the subsequent acidic pH condition (the second phase) for GABA production.

### Analytical Methods

Recombinant *E. coli* cell growth was monitored by measuring optical density at 600 nm (OD_600_) using microplate reader (SpectraMax iD3, Molecular Devices, USA). GABA and glutamate concentrations in the culture broth were determined using a high-performance liquid chromatograph system (1220 Infinity II LC, Agilent Technologies Inc., USA) equipped with a UV detector and ZORBAX SB-C18 column (250 × 4.6 mm) (Agilent Technologies Inc.) as previously reported [19]. Acetate and glucose concentrations were measured by HPLC equipped with an Aminex HPX-76H column (300 × 7.8 mm) (Bio-Rad, USA) and RI detector as previously reported [22].

### Computational Analytical Methods

To analyze how the growth environment affects the metabolic networks associated with GABA production, flux changes induced by each conditional shift were simulated. The target *E. coli* strain was DGB303 (*i.e.*, BL21(DE3) Δ*gabT* Δ*sucA* expressing *icdA*, *gdhA*, *gadBC*, and *dr1558*), and its metabolic network was computationally modeled from the *E. coli* GEM. Among GEMs reconstructed for *E. coli* strains, we used iEC1356_Bl21DE3. This *E. coli* GEM was reconstructed by integrating genomic and physiological data of the *E. coli* BL21 strain. The iEC1356_Bl21DE3 was released in BIGG, containing 1943 metabolites, 2741 reactions, and 1337 genes.

To express the metabolic network of the DGB303 strain, flux values of the two reactions 2-oxoglutarate dehydrogenase (AKGDH) and 4-aminobutyrate aminotransferase (ABTA), which are linked with the *sucA* and *gabT* genes through the GPR (gene-protein-reaction) relationship in the GEM, were set to be zero. This reconstructed GEM was simulated to examine the DGB303 strain’s intracellular metabolic flux distribution when producing GABA under the three different cultivation condition sets: pH shift from neutral to acid (pH 7.0 vs pH 5.0) and succinic acid supplementation (2 g/l vs 4 g/l). Next, to reflect the environmental effects on the DGB303 strain’s metabolic network, constraints for the uptake or secretion rates of five main metabolites (glucose, glutamate, succinic acid, acetic acid, and GABA) and the cell growth rate were obtained from the corresponding fermentation profile’s exponential growth phase under each condition (Table 1).

Parsimonious flux balance analysis (pFBA) and least absolute deviation (LAD) optimization were conducted for GEM simulation [23-25]. The overall process of reconstructing and simulating the GEM was implemented with COBRApy version 0.25.0 [26] and gurobipy version 9.5.1 (Gurobi Optimization, LLC).

## Results and Discussion

### Effect of Succinic Acid Addition and pH Drop on GABA Production

**Fig. 2:**
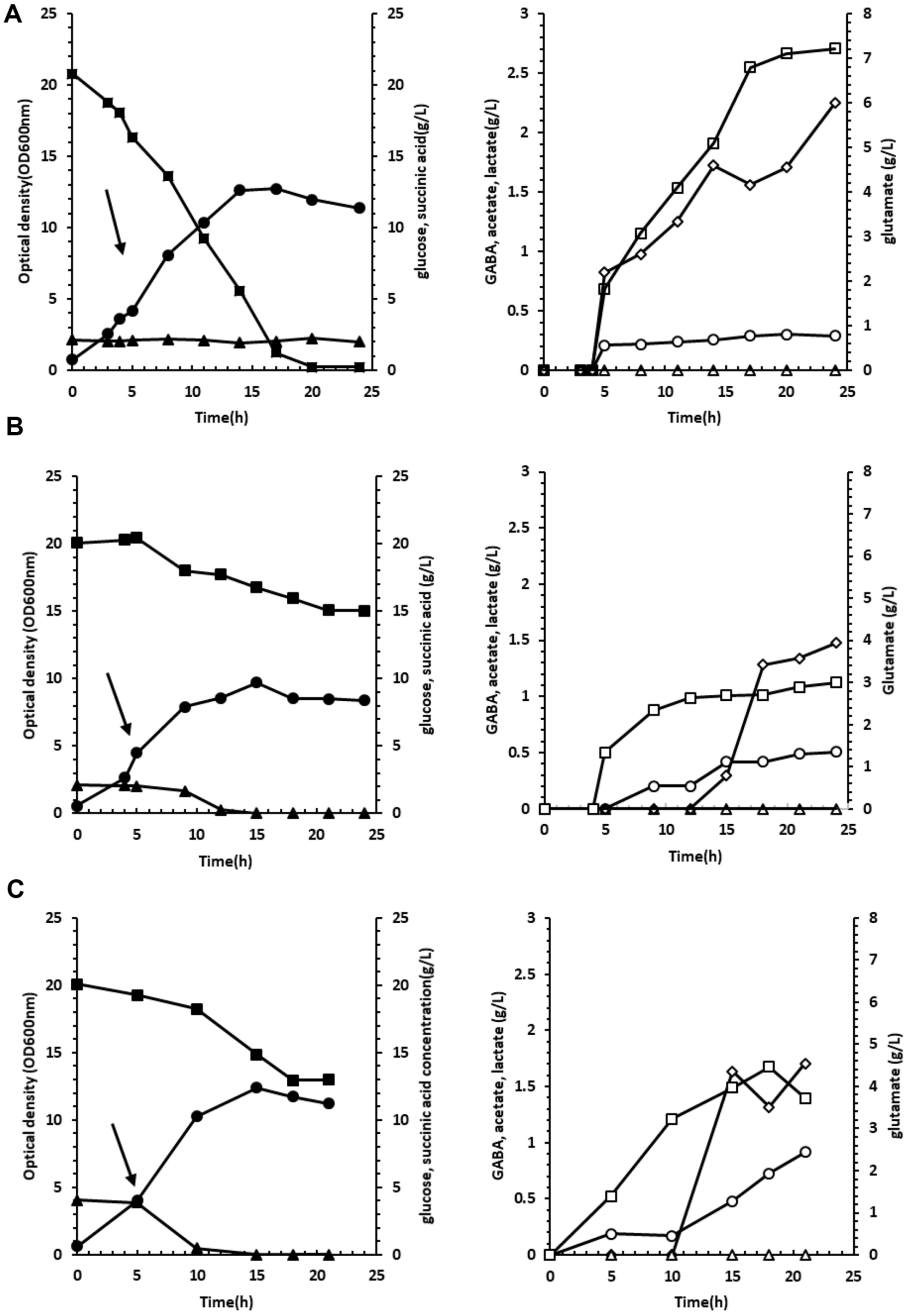
Batch fermentation profiles of the *E. coli* DGB303 strain under three different conditions. (**A**) Batch fermentation profiles at pH 7.0 with 2 g/l succinic acid, (**B**) pH 5.0 after IPTG induction with 2 g/l succinic acid, and (**C**) pH 5.0 after IPTG induction with 4 g/l succinic acid. Except for pH values and succinic acid concentrations, all batch fermentations were conducted in a 1.8 L MR medium with 20 g/l glucose, 10 g/l ammonium sulfate, and 5 g/l yeast extract for 24 h. The black arrow represents induction timing for gene expression. Symbols: filled circle (●), optical density (OD_600_); filled square (■), glucose concentration; filled triangle (▲), succinic acid concentration; open circle (○), GABA concentration; open diamond (◇), acetate concentration; open triangle (△), lactate concentration; open squares (□), glutamate concentration.

Our previous study found that *E. coli* DGB303 (Δ*gabT*, Δ*sucA*, and the overexpression of *icdA*, *gdhA*, *gadB*, *gadC*, and *dr1558*) outperformed *E. coli* DGB203 (Δ*gabT* and the overexpression of *icdA*, *gdhA*, *gadB*, *gadC*, and *dr1558*) concerning GABA production. However, the DGB303 was not subjected to further fed-batch fermentation because of its remarkably low growth rate [19]. Therefore, this strain was subjected to batch fermentation using a 5.0 L fermentor with 1.8 L working volume to reproduce DGB303’s GABA production and growth rate. For this, pH was maintained at 7.0, and the initial dissolved oxygen concentration (DOC) was maintained at 20%. Cell growth (OD_600nm_) peaked at 12.7 h, and glucose was completely consumed by 20 h (Fig. 2A). Glutamate, a precursor of GABA, was produced at 7.21 g/l, but the GABA concentration was only 0.29 g/l after IPTG induction (Fig. 2A).

For the batch fermentation profiles of the DGB303 strain, pH shift and succinic acid supplementation were considered to enhance GABA production and growth. First, because GAD performs best at around a pH of 4.5, dual-phase fermentation was implemented by shifting the pH from neutral to 5.0 upon IPTG induction (Fig. 2B). In general, recombinant *E. coli* does not grow well at pH 5. However, from our previous study, the heterologous expression of *dr1558* improved growth at low pH [19, 22, 27, 28]. Due to the pH shift, GABA concentration increased from 0.29 to 0.51 g/l (Fig. 2B). Despite some promising GABA production results in response to the pH shift, cell growth lowered from an OD_600_ of 11.37 to 8.4 (Fig. 2B).

Because *sucA* was already disrupted in the DGB303 strain, the succinic acid concentration in the medium was doubled from 2 to 4 g/l to restore the TCA cycle fluxes. Indeed, additional succinic acid availability improved cell growth from an OD_600_ of 8.4 to 11.2 (Fig. 2C) and elevated the GABA concentration from 0.51 to 0.92 g/l (Fig. 2C).

### Computational Prediction for Flux Changes Induced by Each Environmental Shift

To systematically analyze the effect of each environmental shift on GABA-producing metabolism in *E. coli* DGB303, batch fermentation profile data before and after the conditional shift were used. For each case, the time interval corresponding to the log phase was specified by selecting the section with the highest slope in the growth curve. Then, exchange fluxes and specific growth rates were calculated for the constraint or reference flux value for pFBA and LAD. As with pFBA, LAD also attempts to accurately predict intracellular flux values [23, 24]. LAD can predict intracellular fluxes in genetically perturbed metabolism via gene knockout. Because *sucA* and *gabT* were genetically perturbed via knockout in this study, LAD was used with pFBA. The object function of pFBA was cell growth. The calculated external fluxes from fermentation results are shown in Table 1. After computational optimization, we compared the flux distributions before and after the shift based on reactions whose predicted flux change ratio was more than 20% to consider significant flux changes only.

**Fig. 3:**
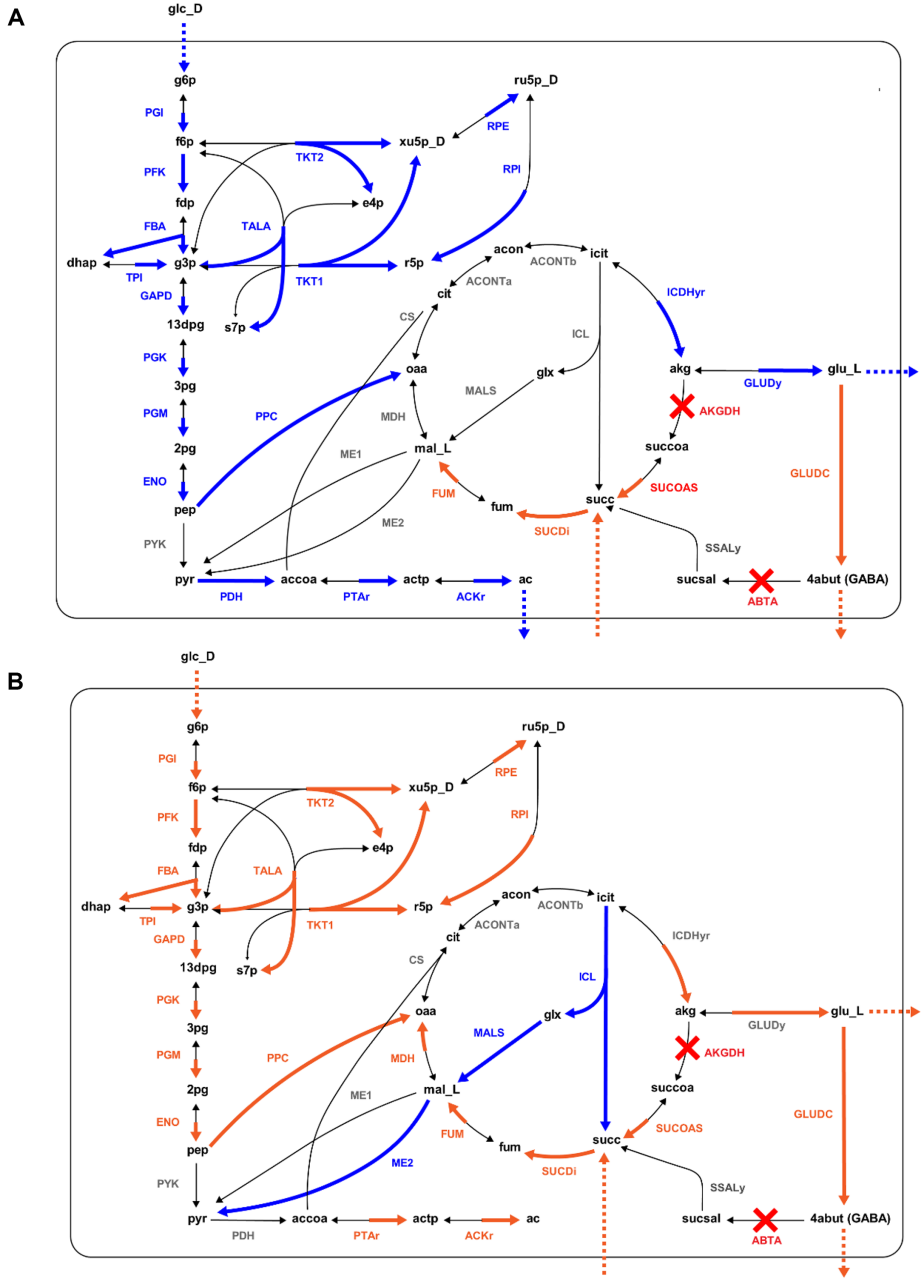
Results of metabolic flux simulated with *E. coli* GEM iEC1356_Bl21DE3. The *E. coli* DGB303 strain was reconstructed by knocking out the AKGDH and ABTA reactions. Reactions in the central metabolic pathways involved in GABA production (glycolysis, pentose phosphate pathway, acetate fermentation, and GABA shunt) were divided into three groups whether their flux was induced, suppressed, or showed no significant change from pFBA and LAD. Results between each condition pair, including pH level between neutral and acidic conditions (**A**) and succinic acid additions of between 2 g/l and 4 g/l (**B**) are represented as a metabolic map. For each figure, orange indicates the induced group, and blue indicates the suppressed group. The reactions included in this figure are presented in Supplementary Table 1.

For pH shift, batch fermentation at neutral (pH 7.0) and acidic states (pH 5.0) were used (Fig. 2A) vs (Fig. 2B). In each profile, the time interval considered as the log phase was 5 h to 8 h for neutral and 5 h to 9 h for acidic conditions. The calculated results of exchange fluxes and cell growth rates had some adjustments based on observation values, but the magnitude relationship before and after the pH shift was unchanged. Concerning internal flux, some distinct changes in flux distributions were predicted due to the pH shift (Fig. 3A). As cell growth and glucose uptake rates were reduced by the pH shift from neutral to acid, the fluxes of many reactions during glycolysis and in the pentose phosphate pathway were also predicted to be reduced under the acidic state. In addition, even though acetic acid production was not apparent in the acidic condition, reaction fluxes in the acetate production pathway were also anticipated to be significantly increased due to the pH shift. These results were consistent with previous studies on metabolic changes in *E. coli* strains under external acidic conditions. For example, James *et al*. proved that the metabolic shift of the *E. coli* strain MG1655 with external acid reduced glucose consumption during cell growth and repressed gene expression, including *ackA* and *pta*. These genes are involved in phosphotransacetylase (PTAr) and acetate kinase (ACKr) reactions, and their repression was also reflected in our prediction as these two reactions were estimated to exhibit decreased flux when the cell grew under acidic conditions [29].

In the TCA cycle, reactions near the succinate, including fumarase (FUM), succinate dehydrogenase (SUCDi), and succinyl-CoA synthetase (SUCOAS), were predicted to be induced by an external acid. In the case of SUCOAS, the reaction direction was predicted to be inverted to produce succinate by pH shift. The acidic induction of the SucC enzyme, which catalyzes the SUCOAS reaction in the *E. coli* strain W3110, has been proven in a previous study [30]. However, James *et al*. also found disruption of the TCA cycle, as the enzymes in the TCA cycle (including *sdhA*) that catalyze the reaction SUCDi are inhibited when *E. coli* grows in low pH. On the other hand, our results predicted that it would be induced. We theorized that this discrepancy is likely because the knockout of 2-oxoglutarate dehydrogenase (AKGDH) in DGB303 made normal TCA circulation infeasible. The subsequent reverse of SUCOAS and the induction of other TCA reactions were derived as a result of optimization to achieve maximal cell growth by directing the TCA cycle from absorbed succinate to 2-oxoglutarate. Regarding reactions related to glutamate and GABA, with reduced glutamate production, isocitrate dehydrogenase (ICDHyr) and glutamate dehydrogenase (GLUDy) showed lowered flux values under acidic conditions. In contrast, as we expected, glutamate decarboxylase (GLUDC) was predicted to have increased flux from acid.

For the next comparison condition, succinic acid addition, the change of metabolic flux was analyzed by comparing the two batch fermentation experiments where the succinic acid concentrations were 2 g/l and 4 g/l (Fig. 2B vs Fig. 2C). The annotated log phase in the profile for each succinic acid concentration condition was 5 h to 9 h for 2 g/l and 5 h to 10 h for 4 g/l. As a result of the flux simulation through pFBA and LAD, the magnitude relationship of the cell growth rate and exchange fluxes before and after succinic acid supplementation was unchanged from the observational results.

When looking into the simulated flux distribution of the internal metabolism (Fig. 3B), the simulation result indicated enhanced reactions in glycolysis and induced reactions in the pentose phosphate pathway following succinic acid addition. With an increased succinate uptake rate, the overall fluxes in the TCA cycle were predicted to be enhanced. Also, starting from the malic enzyme (ME2) that synthesizes pyruvate from L-malate, flux through reactions in the acetate fermentation pathway from pyruvate (including PTAr and ACKr) was predicted to increase in the medium supplemented with succinic acid. However, there was no acetate output of the cell, potentially caused by ME2 suppression. From this, we theorized that higher cell growth rates can be achieved by inducing the TCA cycle and acetate metabolism when the glucose uptake rate is predicted to increase from the additional succinic acid. In the case of GLUDC, its flux was expected to be enhanced after succinic acid addition, based on the simulation results.

Some interesting results were obtained from the internal flux analysis. First, pH shift can increase glutamate decarboxylase (GLUDC) activity, elevating GABA production. However, cultivation at a low pH caused the low growth of host cells. Furthermore, the acetate pathway was not activated, which was probably caused by the inactivation of *sucA*. Notably, adding succinic acid restored cell growth with increased GABA production at a low pH. In particular, fluxes through glycolysis and the pentose phosphate pathway increased at pH 5. This observation indicated that low glycolysis and pentose phosphate pathway fluxes were caused not only by low pH but also by a limiting factor in the TCA pathway. Therefore, succinic acid supplementation may be the solution for the glucose uptake rates in this *sucA* knockout mutant. If the *sucA* gene was properly repressed, high cell growth and GABA production could be obtained without succinic acid addition. Therefore, one of the targets for further development of the GABA producer may be the repression of *sucA*, as supported by a recent report on gene repression using CRISPR-dCas9.

## Conclusion

A previously constructed recombinant *E. coli* strain was cultivated, and its internal flux was simulated in this study. The pH shift from neutral to an acidic pH 5 in broth enhanced GABA production, but poor cell growth was observed. However, adding succinic acid was effective for cell growth without affecting the increased GABA production. Based on the internal flux simulation, it was theorized that the TCA pathway could affect glycolysis and the pentose phosphate pathway. Therefore, proper flux distribution by *sucA* through gene repression could enhance GABA production further.

## Supplemental Materials

Supplementary data for this paper are available on-line only at http://jmb.or.kr.



## Figures and Tables

**Table 1:** Exchange flux of main metabolites and specific growth rates for each pH condition.

Glucose (mmol/gDCW/h)	Succinic acid (mmol/gDCW/h)	GABA (mmol/gDCW/h)	Glutamate (mmol/gDCW/h)	Acetic acid (mmol/gDCW/h)	Growth rate (h^-1^)
Neutral pH, 2 g/l succinic acid					
1.806954	0.086198	0.008443	1.021887	0.306986	0.221086
Acidic pH, 2 g/l succinic acid					
1.201905	0.272554	0.177000	0.611939	0.000000	0.140696
Acidic, 4 g/l succinic acid					
1.8382	1.785084	0.2756	0.773662	0.000000	0.188196

## References

[1] Hayakawa K, Kimura M, Kasaha K, Matsumoto K, Sansawa H, Yamori Y (2004). Effect of a gamma-aminobutyric acid-enriched dairy product on the blood pressure of spontaneously hypertensive and normotensive Wistar-Kyoto rats. Br. J. Nutr..

[2] Adeghate E, Ponery AS (2002). GABA in the endocrine pancreas: cellular localization and function in normal and diabetic rats. Tissue Cell.

[3] Boonstra E, de Kleijn R, Colzato LS, Alkemade A, Forstmann BU, Nieuwenhuis S (2015). Neurotransmitters as food supplements: the effects of GABA on brain and behavior. Front. Psychol..

[4] Sarasa SB, Mahendran R, Muthusamy G, Thankappan B, Selta DRF, Angayarkanni J (2020). A Brief review on the non-protein amino acid, gamma-amino butyric acid (GABA): its production and role in microbes. Curr. Microbiol..

[5] Park SJ, Kim EY, Noh W, Oh YH, Kim HY, Song BG (2013). Synthesis of nylon 4 from gamma-aminobutyrate (GABA) produced by recombinant *Escherichia coli*. Bioprocess Biosyst. Eng..

[6] Yu P, Ma J, Zhu P, Chen Q, Zhang Q (2021). Enhancing the production of γ-aminobutyric acid in *Escherichia coli* BL21 by engineering the enzymes of the regeneration pathway of the coenzyme factor pyridoxal 5'-phosphate. World J. Microbiol. Biotechnol..

[7] Liu Q, Cheng H, Ma X, Xu N, Liu J, Ma Y (2016). Expression, characterization and mutagenesis of a novel glutamate decarboxylase from *Bacillus megaterium*. Biotechnol. Lett..

[8] Masuda K, Guo X, Uryu N, Hagiwara T, Watabe S (2008). Isolation of marine yeasts collected from the Pacific Ocean showing a high production of gamma-aminobutyric acid. Biosci. Biotechnol. Biochem..

[9] Wang J, Lee CL, Pan TM Improvement of monacolin K, gamma-aminobutyric acid and citrinin production ratio as a function of environmental conditions of Monascus purpureus NTU 601. J. Ind. Microbiol. Biotechnol..

[10] Tang CD, Li X, Shi HL, Jia YY, Dong ZX, Jiao ZJ (2020). Efficient expression of novel glutamate decarboxylases and high level production of γ-aminobutyric acid catalyzed by engineered *Escherichia coli*. Int. J. Biol. Macromol..

[11] Luo H, Liu Z, Xie F, Bilal M, Liu L, Yang R, Wang Z (2021). Microbial production of gamma-aminobutyric acid: applications, state-ofthe-art achievements, and future perspectives. Crit. Rev. Biotechnol..

[12] Yuan H, Wang H, Fidan O, Qin Y, Xiao G, Zhan J (2019). Identification of new glutamate decarboxylases from *Streptomyces* for efficient production of γ-aminobutyric acid in engineered *Escherichia coli*. J. Biol. Eng..

[13] Le Vo TD, Kim TW, Hong SH (2012). Effects of glutamate decarboxylase and gamma-aminobutyric acid (GABA) transporter on the bioconversion of GABA in engineered *Escherichia coli*. Bioprocess Biosyst. Eng..

[14] Yu P, Chen K, Huang X, Wang X, Ren Q (2018). Production of γ-aminobutyric acid in *Escherichia coli* by engineering MSG pathway. Prep. Biochem. Biotechnol..

[15] Pham VD, Lee SH, Park SJ, Hong SH (2015). Production of gamma-aminobutyric acid from glucose by introduction of synthetic scaffolds between isocitrate dehydrogenase, glutamate synthase and glutamate decarboxylase in recombinant *Escherichia coli*. J. Biotechnol..

[16] Zhao A, Hu X, Wang X (2017). Metabolic engineering of *Escherichia coli* to produce gamma-aminobutyric acid using xylose. Appl. Microbiol. Biotechnol..

[17] Pham VD, Somasundaram S, Lee SH, Park SJ, Hong SH (2016). Efficient production of gamma-aminobutyric acid using *Escherichia coli* by co-localization of glutamate synthase, glutamate decarboxylase, and GABA transporter. J. Ind. Microbiol. Biotechnol..

[18] Tang CD, Li X, Shi HL, Jia YY, Dong ZX, Jiao ZJ (2020). Efficient expression of novel glutamate decarboxylases and high level production of γ-aminobutyric acid catalyzed by engineered *Escherichia coli*. Int. J. Biol. Macromol..

[19] Park SH, Sohn YJ, Park SJ, Choi J (2020). Effect of DR1558, a *Deinococcus radiodurans* response regulator, on the production of GABA in the recombinant *Escherichia coli* under low pH conditions. Microb. Cell Fact..

[20] Nielsen J (2017). Systems biology of metabolism. Annu. Rev. Biochem..

[21] Orth JD, Thiele I, Palsson BØ (2010). What is flux balance analysis?. Nat. Biotechnol..

[22] Kang S, Choi J (2021). Enhanced cadaverine production by recombinant *Corynebacterium glutamicum* with a heterologous DR1558 regulator at low pH condition. Process Biochem..

[23] Lee SM, Lee GR, Kim HU (2022). Machine learning-guided evaluation of extraction and simulation methods for cancer patientspecific metabolic models. Comput. Struct. Biotechnol. J..

[24] Lewis NE, Hixson KK, Conrad TM, Lerman JA, Charusanti P, Polpitiya AD (2010). Omic data from evolved *E. coli* are consistent with computed optimal growth from genome-scale models. Mol. Syst. Biol..

[25] Lee D, Smallbone K, Dunn WB, Murabito E, Winder CL, Kell DB (2012). Improving metabolic flux predictions using absolute gene expression data. BMC Syst. Biol..

[26] Ebrahim A, Lerman JA, Palsson BØ, Hyduke DR (2013). COBRApy: constraints-based reconstruction and analysis for python. BMC Syst. Biol..

[27] Park S, Kim GB, Kim HU, Park SJ, Choi J (2019). Enhanced production of poly-3-hydroxybutyrate (PHB) by expression of response regulator DR1558 in recombinant *Escherichia coli*. Int. J. Biol. Macromol..

[28] Kang SB, Choi J (2022). Production of cadaverine in recombinant *Corynebacterium glutamicum* overexpressing lysine decarboxylase (ldcC) and response regulator dr1558. Appl. Biochem. Biotechnol..

[29] Orr JS, Christensen DG, Wolfe AJ, Rao CV (2018). Extracellular acidic pH inhibits acetate consumption by decreasing gene transcription of the tricarboxylic acid cycle and the glyoxylate shunt. J. Bacteriol..

[30] Stancik LM, Stancik DM, Schmidt B, Barnhart DM, Yoncheva YN, Slonczewski JL (2002). pH-dependent expression of periplasmic proteins and amino acid catabolism in *Escherichia coli*. J. Bacteriol..

