# Supplementary material for: Intracellular Flux Prediction of Recombinant Escherichia coli Producing Gamma-Aminobutyric Acid
Source: J Microbiol Biotechnol. 2024 Jan 30;34(4):978–84. doi: 10.4014/jmb.2312.12022 (PMC11091657; doi:10.4014/jmb.2312.12022)
Supplement: Supplementary file 1 [file jmb-34-4-978-supple.pdf]

J. Microbiol. Biotechnol.
https://doi.org/10.4014/jmb.2312.12022

Supplementary Table 1. Reaction list shown in Figure 3.

Reaction ID	Reaction name	Reaction	Pathway
PGI	Glucose-6-phosphate isomerase	g6p_c → f6p_c	glycolysis
PFK	Phosphofructokinase	atp_c + f6p_c → adp_c + fdp_c + h_c	glycolysis
TPI	Triose-phosphate isomerase	dhap_c → g3p_c	glycolysis
GAPD	Glyceraldehyde-3-phosphate dehydrogenase	g3p_c + nad_c + pi_c → 13dpg_c + h_c + nadh_c	glycolysis
PGK	Phosphoglycerate kinase	13dpg_c + adp_c →3pg_c + atp_c	glycolysis
PGM	Phosphoglycerate mutase	3pg_c → 2pg_c	glycolysis
ENO	Enolase	2pg_c → h2o_c + pep_c	glycolysis
FBA	Fructose-bisphosphate aldolase	fdp_c → dhap_c + g3p_c	glycolysis
RPE	Ribulose 5-phosphate 3-epimerase	xu5p__D_c → ru5p__D_c	pentose phosphate pathway
TKT2	Transketolase	f6p_c + g3p_c → xu5p__D_c + e4p_c	pentose phosphate pathway
RPI	Ribose-5-phosphate isomerase	ru5p__D_c → r5p_c	pentose phosphate pathway
TALA	Transaldolase	e4p_c + f6p_c →g3p_c + s7p_c	pentose phosphate pathway
TKT1	Transketolase	g3p_c + s7p_c → xu5p__D_c + r5p_c	pentose phosphate pathway

GLUDy	Glutamate dehydrogenase (NADP)	akg_c + h_c + nadph_c + nh4_c → glu__L_c + h2o_c + nadp_c	L-glutamate biosynthesis
PPC	Phosphoenolpyruvate carboxylase	co2_c + h2o_c + pep_c → h_c + oaa_c + pi_c	mixed acid fermentation
PDH	Pyruvate dehydrogenase	coa_c + nad_c + pyr_c → accoa_c + co2_c + nadh_c	pyruvate fermentation to acetate
PTAr	Phosphotransacetylase	accoa_c + pi_c → actp_c + coa_c	pyruvate fermentation to acetate
ACKr	Acetate kinase	actp_c + adp_c → ac_c + atp_c	pyruvate fermentation to acetate
ICDHyr	Isocitrate dehydrogenase (NADP)	icit_c + nadp_c → akg_c + co2_c + nadph_c	TCA cycle
AKGDH	2-Oxogluterate dehydrogenase	akg_c + coa_c + nad_c → co2_c + nadh_c + succoa_c	TCA cycle
FUM	Fumarase	fum_c + h2o_c → mal__L_c	TCA cycle
SUCOAS	Succinyl-CoA synthetase	adp_c + pi_c + succoa_c → atp_c + coa_c + succ_c	TCA cycle
SUCDi	Succinate dehydrogenase	q8_c + succ_c → fum_c + q8h2_c	TCA cycle
GLUDC	Glutamate Decarboxylase	glu__L_c + h_c → 4abut_c + co2_c	GABA shunt
ABTA	4-aminobutyrate transaminase	4abut_c + akg_c → glu__L_c + sucsal_c	GABA shunt

EX_succ_e*	Succinate exchange	succ_e → succ_c
EX_glc__D_e	D-Glucose exchange	glc__D_e → glc__D_c
EX_succ_e	Succinate exchange	succ_c → succ_e
EX_glu__L_e	L-Glutamate exchange	glu__L_c → glu__L_e
EX_ac_e	Acetate exchange	ac_c → ac_e
EX_4abut_e	4-Aminobutanoate exchange	4abut_c → 4abut_e

Supplementary Table 2. List of simulated flux values for reactions under each condition.

Reaction ID	Flux under neutral pH	Flux under acidic pH with 2 g/L succinic acid	Flux under acidic pH with 4 g/L succinic acid
PGI	2.839207	1.721632	2.918226
RPE	0.137229	0.087331	0.151215
TKT2	0.102338	0.065127	0.113513
RPI	0.130188	0.082850	0.151312
PFK	1.767253	0.844632	1.813455
FBA	1.767253	0.844632	1.813455
TALA	0.077045	0.049031	0.075133
TKT1	0.034891	0.022204	0.031542
TPI	2.660274	1.607761	2.575651
GAPD	5.252755	3.172380	5.12648
ICDHyr	1.220499	0.909959	1.210531
GLUDy	2.543077	1.751632	2.351357
PGK	5.252755	3.172380	5.153141
PGM	4.948647	2.978849	4.913453
ENO	4.948647	2.978849	4.913453
PPC	1.086968	0.411707	1.0215789
PDH	3.193629	2.142003	2.313503
PTAr	0.372242	0.041579	0.351355

ACKr	0.372242	0.041579	0.356126
EX_glc__D_e	2.839207	1.721632	2.788226
EX_succ_e	0.086198	0.272554	1.785084
EX_glu__L_e	1.021887	0.611939	0.773662
EX_ac_e	0.306986	0	0
FUM	0.651228	0.827708	0.913134
SUCOAS*	-0.092831	78.504992	85.32574
SUCDi	1.178877	1.593584	1.721256
GLUDC	0.008443	0.177000	0.275612
EX_succ_e*	0.086198	0.272554	1.785084
EX_4abut_e	0.008443	0.177000	0.275612
